# Post-mortem computed tomography coaxial cutting needle biopsy to facilitate the detection of bacterioplankton using PCR probes as a diagnostic indicator for drowning

**DOI:** 10.1007/s00414-016-1473-6

**Published:** 2016-11-05

**Authors:** Guy N. Rutty, Christopher Johnson, Jasmin Amoroso, Claire Robinson, Carina J. Bradley, Bruno Morgan

**Affiliations:** 1 East Midlands Forensic Pathology Unit, University of Leicester, Robert Kilpatrick Building, Leicester, LE2 7LX UK; 2 Imaging Department, University Hospitals of Leicester, Leicester Royal Infirmary, Leicester, LE2 7LX UK; 3 Clinical Microbiology, Empath Pathology Services, University Hospitals of Leicester NHS Trust, Leicester Royal Infirmary, Leicester, LE1 5WW UK; 4 Radiology Department, University of Leicester, Robert Kilpatrick Building, Leicester, LE2 7LX UK

**Keywords:** Forensic, Post-mortem computed tomography, PMCT, Coaxial cutting needle biopsy, Drowning, Bacterioplankton, Real-time PCR

## Abstract

We report for the first time the use of coaxial cutting needle biopsy, guided by post-mortem computed tomography (PMCT), to sample internal body tissues for bacterioplankton PCR analysis to investigate drowning. This technical report describes the biopsy technique, the comparison of the needle biopsy and the invasive autopsy sampling results, as well as the PMCT and autopsy findings. By using this new biopsy sampling approach for bacterioplankton PCR, we have developed on previous papers describing the minimally invasive PMCT approach for the diagnosis of drowning. When such a system is used, the operator must take all precautions to avoid contamination of the core biopsy samples due to the sensitivity of PCR-based analytic systems.

## Introduction

Post-mortem cross-sectional imaging is playing an increasing role as a diagnostic adjunct or a replacement to invasive autopsy, and a number of authors have drawn attention to the post-mortem computed tomography (PMCT) observations associated with drowning. They have reported fluid within the paranasal sinuses, fluid within the airways, chest and abdominal structures, changes to the lung parenchyma and the presence of haemodilution observed in the right and left atria and spleen [1–10]. Although Raux et al. have suggested an algorithm to diagnose drowning on PMCT [4], the findings of PMCT remain non-specific [9], and therefore, PMCT remains only an adjunct to invasive autopsy in suspected drowning.

The concept of using polymerase chain reaction (PCR) methods to amplify bacteria genes to aid the diagnosis of drowning was originally reported by Suto et al. in 2007 [11]. In 2012, Uchiyama et al. reported the use of real-time PCR assays with TaqMan probes for bacterioplankton to differentiate between freshwater and marine bacterioplankton DNA and provide a molecular diagnostic test to assist in the diagnosis of drowning [12]. More recently, Rutty et al. reported their experience of the use of bacterioplankton PCR probes for the diagnosis of drowning within England [13]. The diagnostic material required for the PCR tests has previously been obtained through invasive autopsy.

We propose that these samples could be obtained using coaxial cutting needle biopsies guided by PMCT [14]. In this case report, we demonstrate the successful use of this technique for the first time in order to help other teams who are using PMCT to investigate bodies discovered immersed in water. We discuss the potential for DNA contamination during coaxial PMCT needle biopsy sampling.

## Materials and method

### Case summary

An 81-year-old male with a known history of depression and previous attempted suicide by slashing his wrists and hanging, took a taxi from his home to a fairly remote location near to a river. He failed to return home, and on the 8th day, a body was located, caught up in foliage overhanging the swollen river. The body was subsequently identified to be the missing male. A medico-legal autopsy was authorised by Her Majesty’s Senior Coroner for the jurisdiction of the death. This was undertaken 6 days after the discovery of the body (14 days after the disappearance). On the day prior to the autopsy, the body underwent PMCT examination.

The deceased was consented for PMCT research, including core needle biopsy, pulmonary ventilation and targeted limb vessel angiography in accordance with previously published ethics and consenting procedures [15] by the relatives of the deceased. The ventilation and angiography procedures were undertaken following core needle biopsy sampling and do not form part of this report.

### PMCT needle biopsy samples

Prior to needle biopsy, a whole body PMCT scan was performed using a Toshiba Aquilion CXL 128 slice scanner (120 kVp, 300 mA and 128 × 0.5-mm slice thickness, matrix 512 × 512) reconstructed to 1 mm (head and neck) or 2 mm (chest, abdomen, pelvis and legs) slices.

Cutting needle biopsies were undertaken using a technique similar to clinical radiology practice [14]. A co-axial technique was used to avoid cellular contamination of the biopsy needle from the skin. A new needle was used for each biopsy (16G Coaxial Temno Evolution biopsy needle, Carefusion, McGaw Park, IL, USA). Ten-millimetre-long samples were taken, in order from the left lung, spleen, left kidney and brain.

**Fig. 1:**
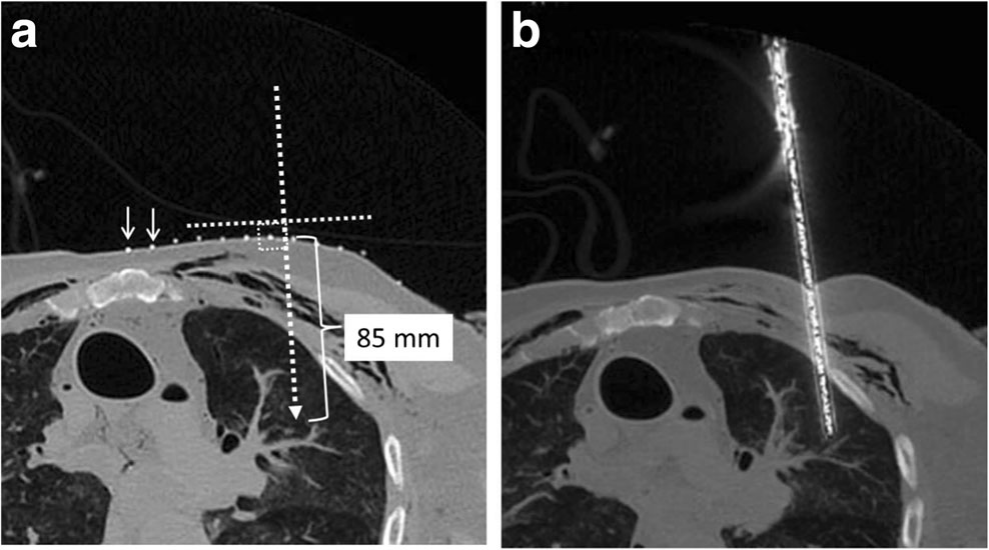
**a** The radio-opaque skin marker strip is placed on the left chest wall (*small arrows*), and the angle of approach and depth of insertion of the needle can be planned using the geometry tools on the CT workstation. The needle is then be inserted at the planned angle and distance and a further set of CT images (**b**) are acquired to confirm the location of the needle prior to biopsy

**Fig. 2:**
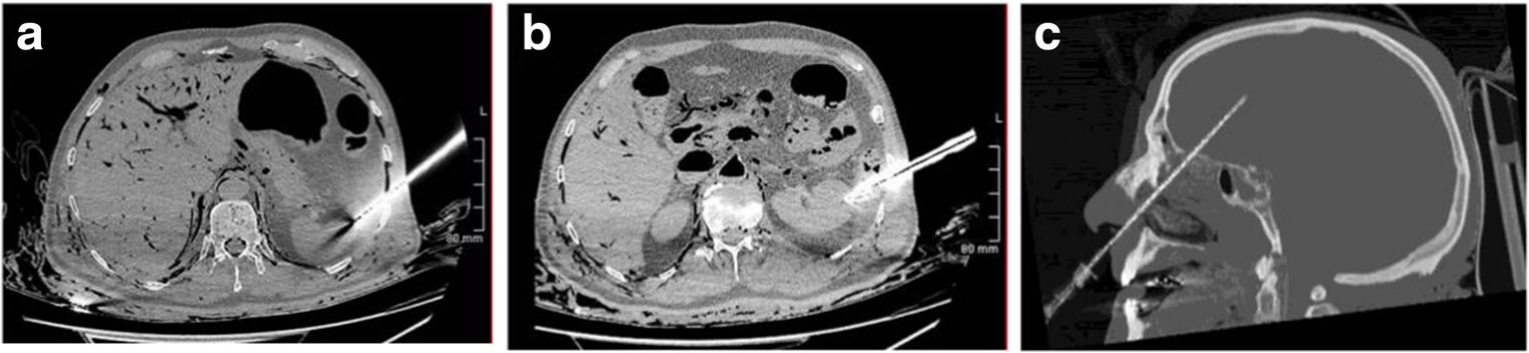
PMCT images of the angle and position of the cutting needle prior to sampling of the **a** spleen, **b** left kidney and **c** brain

The first sample obtained was from the left lung. The desired biopsy location was selected from the images, and a radio-opaque skin marker strip was placed on the adjacent chest wall after cleaning with alcohol. The geometry tools on the CT workstation were used to determine the angle of approach and depth of insertion of the needle from the skin marker (Fig. 1a). The pathologist then inserted the needle using these parameters (Fig. 1b). Prior to sampling, the needle position was checked by PMCT. Cutting needle biopsy was then performed. The same procedure was then repeated for the spleen (Fig. 2a) and the left kidney (Fig. 2b). In the case of the spleen, the operator should access the inferior aspect of the spleen or insert the needle at an oblique angle to avoid crossing the pleura.

The brain was sampled by inserting the needle through the right nasal cavity at an approximate angle of 45°. This enabled the needle to breach the right cribriform plate and pass into the substance of the right cerebral hemisphere. Again, prior to sampling, the position of the needle was checked on PMCT (Fig. 2c).

All samples were placed into separate sterile containers with no preservative. These were placed into a refrigerator at 4 °C before being processed in the Clinical Microbiology laboratory at Leicester Royal Infirmary.

### Reference water sample

A reference river water sample was obtained from the point where the body was recovered. This was provided to the Clinical Microbiology laboratory with the tissue samples.

### Autopsy

A full invasive autopsy was undertaken the following day by a senior trainee forensic pathologist working to national forensic pathology guidelines [16]. Repeat samples for bacterioplankton PCR were taken from the approximate area of the core needle biopsy sites within the brain, left lung, left kidney and spleen at autopsy. For each case, a new scalpel blade and a clean, different pair of forceps were used for sampling and tissue handling. All samples were again placed into separate sterile universal containers with no preservative. These were placed into a refrigerator at 4 °C before being processed in the Clinical Microbiology laboratory at Leicester Royal Infirmary.

### DNA isolation, PCR investigation and assay analytical sensitivity and linearity

The bacterioplankton PCR procedures used for the investigation of this case followed the methods and materials previously published by Rutty et al. [13]. The 250 μL of sterile phosphate-buffered saline (PBS), as previously added directly to the Roche Green Beads tubes, was used to suspend the needle biopsy material and rinse any debris from the inside of each sample container. This was then added to the green beads tubes along with 250 μL of Roche Bacteria Lysis Buffer before starting the mechanical lysis step and continuing with the DNA extraction and PCR as described in Rutty et al. [13]. For this case, the needle biopsy samples were processed before processing the autopsy tissues and water sample in order to eliminate the possibility of cross-over contamination of the biopsies.

## Results

The results from the needle biopsy and autopsy-derived bacterioplankton PCR analysis are shown in Table 1. This illustrates that for all needle biopsy sites, all three target freshwater bacteria genes were identified. Similar results were observed for the autopsy-derived lung, spleen and brain samples with only one of the genes being identified from the autopsy left kidney and reference water samples.

**Table 1:** DNA PCR results from the river, needle biopsy and autopsy-derived tissue samples for freshwater and marine bacterioplankton DNA

Sample type	Freshwater bacterioplankton DNA	Marine bacterioplankton DNA
Brain tissue	*Aeromonas* sp. DNA detected in all three target genes (*aerA*, *gyrB* and *chiA*)	Not detected by PCR
Kidney tissue	Weakly positive for one of the three *Aeromonas* sp. DNA gene targets, *gyrB* only	Not detected by PCR
Spleen tissue	*Aeromonas* sp. DNA detected in all three target genes (*aerA*, *gyrB* and *chiA*)	Not detected by PCR
Lung tissue	*Aeromonas* sp. DNA detected in all three target genes (*aerA*, *gyrB* and *chiA*)	Not detected by PCR
Water from the river	Weakly positive for one of the three *Aeromonas* sp. DNA gene targets, *gyrB* only	Not detected by PCR
Needle biopsy Brain	*Aeromonas* sp. DNA detected in all three target genes (*aerA*, *gyrB* and *chiA*)	Not detected by PCR
Needle biopsy Left kidney	*Aeromonas* sp. DNA detected in all three target genes (*aerA*, *gyrB* and *chiA*)	Not detected by PCR
Needle biopsy Spleen	*Aeromonas* sp. DNA detected in all three target genes (*aerA*, *gyrB* and *chiA*)	Not detected by PCR
Needle biopsy Lung	*Aeromonas* sp. DNA detected in all three target genes (*aerA*, *gyrB* and *chiA*)	Not detected by PCR

**Fig. 3:**
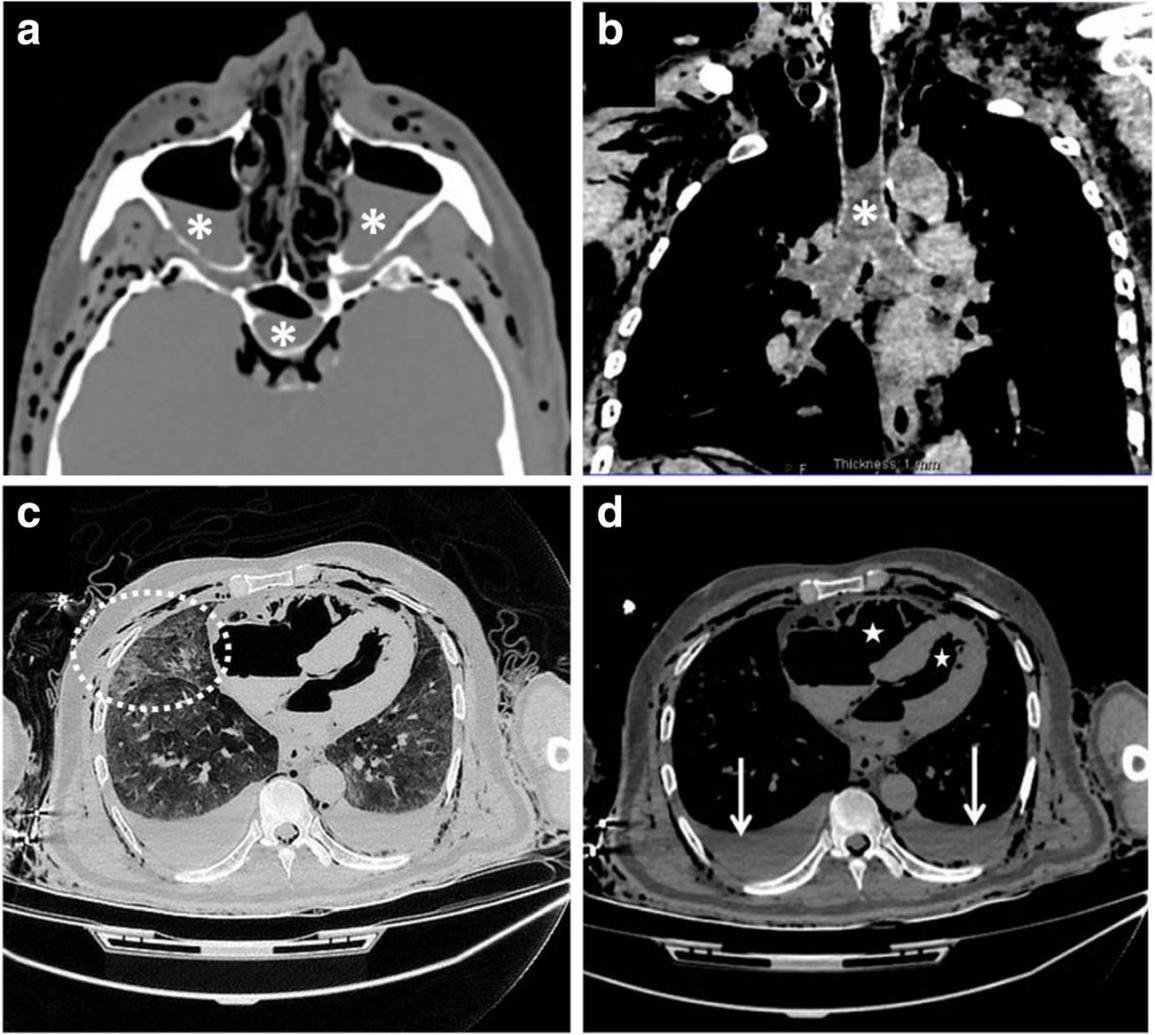
PMCT changes. **a** Fluid within the maxillary and sphenoid sinuses (*asterisks*). **b** Fluid within the trachea (*asterisk*) and bronchi. **c** Ground-glass opacification in the lungs (*within circle*). **d** Pleural effusions (*arrows*) and air in the cardiac chambers (*stars*)

The PMCT examination (Fig. 3) identified fluid within the paranasal sinuses, lower trachea and bronchi as well as in both pleural cavities. The lungs showed mild ground-glass changes, and the stomach contained low density fluid consistent with water. The right and left atria showed gas distension due to decomposition, so it could not be assessed for heamodilution effects. These features are therefore consistent with drowning, but non-specific and could occur in any PMCT scan.

The autopsy identified external changes appropriate for the time between the deceased disappearance and the autopsy, taking into account the time of year (January) and refrigerated body storage. The body and clothing were all wet at scanning and autopsy. The soles of the feet and palms of the hands showed “washerwoman” changes. The hands and forearms were dirty with silt, and there was silt and dirt staining to the front of the thorax and abdomen. There were no external or internal injuries that could have caused or contributed to death. The middle ear areas showed haemorrhage. The lungs contained fluid as did the stomach. One hundred millimetres of fluid were present to each pleural cavity. There was no evidence of natural disease that could have caused or contributed to death. The toxicology examination identified that there has been social level alcohol and therapeutic range citalopram used prior to death. The histology examination showed changes to the lung alveoli consistent with air trapping.

Death was concluded to be as a result of drowning.

## Discussion

The use of cutting needle biopsies with PMCT for diagnostic histological sampling has been previously proposed, principally by the “Virtopsy®” group in Switzerland who, in more recent times, have promoted the use of robotic sampling systems [17–19]. However, in clinical radiology, needle sampling of the tissues of the body for diagnostic purposes is well established. Through this report, we have shown for the first time how such a technique can be used with PMCT for the purposes of providing a minimally invasive approach to the investigation of drowning. The manual sampling was undertaken by a pathologist under the guidance of an interventional radiologist. The training required was minimal to enable accurate organ sampling. We have subsequently trained a radiographer to undertake PMCT-guided cutting needle biopsy.

In this case, four separate isolated organ locations were sampled, i.e. the cranial cavity, left chest cavity, intra- and retro-peritoneal. Unlike at traditional invasive autopsy where the cavities must be opened and organs identified and handled prior to an attempt to undertake sterile sampling [20], the use of PMCT cutting needle biopsies allows for targeted organ sampling. The use of a new needle on each occasion is critical to ensure that cross-contamination does not occur between samples. Manual serial percutaneous biopsies facilitate such an approach. For the autopsy tissue samples, a small section of each tissue is cut for the DNA extraction process. The use of needle biopsies for the bacterioplankton PCR assay also reduced the need to manipulate the tissues, reducing hands-on time in the laboratory and reducing the risk of cross-over contamination during preparation of the samples.

Although a period of time had elapsed between the disappearance and the sampling of the body at PMCT and autopsy, Kakizaki et al. have previously demonstrated that post-mortem bacterial invasion of tissues does not readily occur, even in highly decomposed bodies [21]. Also, although the surface of the body had been exposed to the water, and would be expected to be contaminated with freshwater bacterioplankton, cleaning the skin with alcohol and the use of the coaxial needle, which has been shown to allow clinical sampling of tumours without tumour seeding [22], allowed for organ sampling without the introduction of contaminant from the skin surface during the insertion and withdrawal of the needle.

The question does arise “could contamination of the sample arise because the coaxial system passes through skin surface, water and other tissues prior to reaching the target organ, or as the introducer stylet is removed prior to the sampling”. Currently, the answer to this question is unknown. However, considering the design of the coaxial system, we would not expect contamination to occur. The co-axial system is inserted with an introducer blocking the central channel. After introduction, this introducer needle is withdrawn. The sampling needle is then inserted so the cutting end extends beyond the previous introducer tip position into the target tissue. The mechanism then advances the bladed needle further into the tissue, the sheath then advances over it cutting a sample, which is be contained within the sampling sheath making a “sterile chamber”. As the sampling needle is withdrawn, this tissue remains within this “chamber”. The final sample is removed. We do not immerse the needle tip into the tissue-sample preservative fluid, so only the cut sample is added. In this single case, we have shown that the needle biopsy results are comparable to autopsy-derived samples. The observation that the river water sample does not contain all three bacterioplankton can be hypothesised due to differences between the water bacterioplankton populations at the place and time the deceased actually drowned and the sample was taken. Another hypothesis is that bacterioplankton are more dilute free in water whereas the organisms become more concentrated when filtered into the body via the lungs.

This is only a single-case observation, and we accept that there is an unknown risk that due to the use of PCR, bacterioplankton DNA contamination may occur during the sampling process. A larger study is now underway to validate this technique and to consider the issue of potential contamination, which would have wider implications for all PMCT needle biopsy sampling. However, we feel that it is appropriate to present these results and discussion of potential limitations to autopsy and imaging practitioners for consideration and research.

## Conclusions

We present a single case report to illustrate a new diagnostic technical approach of minimally invasive PMCT enhanced by coaxial cutting needle biopsy sampling for bacterioplankton for the diagnosis of drowning. This paper enhances the previous works related to PMCT and drowning. It also raises a risk for those engaged in PMCT biopsy sampling, i.e. sample contamination. By translating CT-guided biopsy method from clinical radiology, we illustrate a simple method which can be undertaken for diagnostic organ sampling for PCR for the diagnosis of drowning.
